# SGLT2 Inhibitors, Muscle Loss, and Creatinine-Based Estimated GFR: An Integrative Conceptual Review of Renoprotection

**DOI:** 10.1016/j.xkme.2026.101297

**Published:** 2026-02-13

**Authors:** Dion Groothof, Naser B.N. Shehab, Adrian Post, Nicole S. Erler, Reinold O.B. Gans, Stephan J.L. Bakker

**Affiliations:** 1 Department of Internal Medicine, Division of Nephrology, University Medical Center Groningen, University of Groningen, Groningen, the Netherlands; 2 Department of Data Science and Biostatistics, Julius Center for Health Sciences and Primary Care, University Medical Center Utrecht, Utrecht, the Netherlands

**Keywords:** Chronic kidney disease, confounding, creatinine, glomerular filtration rate, methodological bias, muscle mass, SGLT2 inhibitors

## Abstract

Diabetes mellitus profoundly affects the kidneys, driving many individuals to kidney failure. With the rising global incidence of diabetic kidney disease straining health care systems, effective interventions are imperative. Landmark trials have shown that sodium–glucose cotransporter 2 (SGLT2) inhibitors attenuate the decline in estimated glomerular filtration rate (GFR), even without diabetes. However, these trials largely relied on creatinine-based GFR estimates, which are only valid if an intervention does not alter muscle-derived creatinine generation. Because SGLT2 inhibitors reduce muscle mass, the observed attenuation in estimated GFR decline may partly reflect reduced creatinine generation rather than true preservation of GFR. This methodological bias could also skew hard endpoints such as dialysis initiation, transplantation, and mortality. Using representative data from a landmark SGLT2 inhibitor trial, we show that a physiologically plausible reduction in muscle mass could account for the observed preservation of estimated GFR, underscoring the need for careful reinterpretation. The hypothesis that therapy-induced muscle loss contributed to the observed kidney benefits can be tested using directly measured GFR or, alternatively, cystatin C-based GFR estimates. Future modeling studies integrating dual GFR assessments with objective measures of muscle mass and strength are essential to disentangle genuine renoprotection from artifacts arising from creatinine-based GFR estimation.

## Introduction

The prevalence of diabetes is projected to escalate to 1.31 billion cases by 2050.^1^ Approximately 40% will develop diabetic kidney disease, with many progressing to kidney failure, underscoring the need for effective treatments. Results from trials on sodium/glucose cotransporter 2 (SGLT2) inhibitors are unprecedented in this context, offering renoprotection and reduced mortality, even in the absence of diabetes.^2^^,^^3^ These findings prompted rapid approval of SGLT2 inhibitors by regulators in Europe and the United States as treatment for chronic kidney disease (CKD) irrespective of comorbid type 2 diabetes. Nonetheless, circumstantial evidence shows that SGLT2 inhibition reduces muscle mass.^4^^,^^5^ An overview of studies that empirically investigated the effects of SGLT2 inhibitors on muscle mass is summarized elsewhere.^5^ Notably, those studies that measured height-indexed appendicular lean mass—the recommended proxy for muscle mass^6^—consistently reported significant decreases with SGLT2 inhibitors compared with placebo.^5^ We hypothesize that therapy-related muscle loss has confounded the observed renoprotection of SGLT2 inhibitors. This article substantiates this hypothesis by reviewing the metabolism of creatine and creatinine, exploring the metabolic consequences of SGLT2 inhibition, and detailing how these processes reduce muscle mass and affect creatinine concentrations. We then contrast these chronic effects with the acute, muscle mass-independent effects of SGLT2 inhibitors. In addition to conceptual synthesis, we also provide a quantitative estimate of the muscle mass loss that would be required to meaningfully bias creatinine-based estimates of glomerular filtration rate (GFR), offering an illustrative example of the potential magnitude of this effect. Finally, we discuss how changes in muscle mass could have confounded renoprotective effects in landmark trials and conclude with recommendations for future research.

## Creatine and Creatinine Metabolism

**Figure 1:**
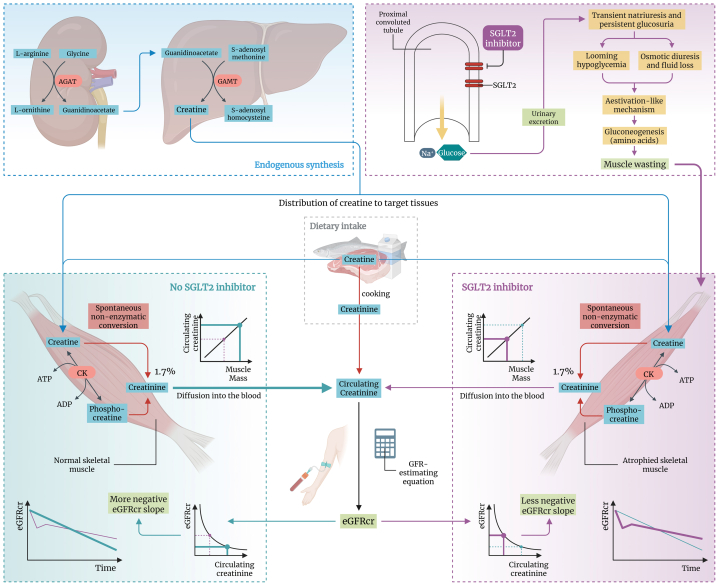
Proposed mechanism underlying the observed renoprotection by SGLT2 inhibitors. We propose that the attenuation in the eGFRcr slope as observed in individuals who received SGLT2 inhibitors compared with those who received placebo in landmark trials was an epiphenomenon of therapy-related loss of muscle mass. Persistent glucosuria because of SGLT2 inhibition activates an aestivation-like water-conserving response that intensifies gluconeogenesis to offset looming hypoglycemia and glucose-driven osmotic diuresis. In this process, amino acids from skeletal muscle proteins are mobilized and degraded to form new glucose molecules and provide the necessary nitrogen for urea generation and accumulation to enable water conservation.^4^ SGLT2 inhibitor therapy causes loss of muscle mass, leading to a reduction in the total creatine pool, which is accompanied by lower creatinine production and release and, consequently, creatinine concentration. Because of the inversely proportional relationship between creatinine concentration and eGFRcr, therapy-related loss of muscle mass causes an upward bias in eGFRcr, irrespective of actual changes in GFR. This spuriously suggests preservation of eGFRcr over time in individuals who received SGLT2 inhibitors compared with those who received placebo. Abbreviations: AGAT, l-arginine:glycine amidinotransferase; CK, creatine kinase; eGFRcr, creatinine-based estimated glomerular filtration rate; GAMT, *S*-adenosyl-l-methionine:*N*-guanidinoacetate methyltransferase; SGLT2, sodium-glucose cotransporter 2. Created with BioRender.com.

The biosynthesis of creatine begins in the kidneys, where l-arginine:glycine amidinotransferase catalyzes the production of guanidinoacetate. This process continues in the liver, where *S*-adenosyl-l-methionine:*N*-guanidinoacetate methyltransferase donates a methyl group from *S*-adenosylmethionine to guanidinoacetate, resulting in creatine formation and distribution to target tissues (Fig 1).^7^ There, creatine kinase catalyzes the transfer of the γ-phosphate of adenosine triphosphate (ATP) to creatine, forming adenosine diphosphate and phosphocreatine, creating a reservoir for instant ATP regeneration during intense activity. However, the system’s sustainability is challenged by the degradation of creatine and phosphocreatine to creatinine at a rate of ∼1.7% daily, depending on temperature and acidity.^7^ This degradation necessitates continued creatine replenishment through diet and endogenous synthesis. Importantly, it also directly links muscle mass to blood creatinine concentrations (Fig 1). As creatinine diffuses into the bloodstream, it is primarily cleared through glomerular filtration, causing steady-state concentrations to be inversely proportional to the GFR. This relationship makes creatinine an adequate proxy of GFR, provided that its relationship with muscle mass is adequately accounted for. In practice, this is done with GFR-estimating equations, which incorporate age and sex to account for variability in creatinine concentrations related to muscle mass,^8^ thus providing a creatinine-based estimated GFR (eGFRcr) that more accurately reflects true GFR than creatinine concentrations alone. Importantly, these equations assume typical muscle mass for a given age and sex.^9^ When this assumption does not hold, eGFRcr may be inaccurate.^8^^,^^10^

## Metabolic Consequences of SGLT2 Inhibition

Physiologically, glucose is freely filtered at the glomerulus and then completely reabsorbed in the proximal tubule.^11^^,^^12^ Reabsorption occurs against a concentration gradient powered by a sodium ion concentration maintained by the sodium–potassium pump. This mechanism creates an energy reservoir to facilitate glucose reabsorption. SGLTs are carrier proteins that bind both sodium and glucose. Once bound, the sodium ion gradient propels their cotransport into the cell. More than 90% of the glucose is reabsorbed into epithelial cells of segment 1 of the proximal convoluted tubule via SGLT2, whereas the remainder is reabsorbed via SGLT1 in segments 2 and 3.^12^ Inhibiting SGLT2 therefore results in natriuresis and glucosuria (Fig 1). Initial increases in sodium excretion are transient because of compensatory sodium reabsorption further down the nephron, whereas persistent glucosuria causes substantial daily glucose losses of ∼75 g (equating to ∼300 kcal), reducing plasma glucose concentrations.^11^^,^^13^ Remarkably, the initial glucose-driven osmotic-diuretic effect is short-lived and looming hypoglycemia is averted, both through intensified gluconeogenesis. These phenomena are hypothesized to be because of a compensatory aestivation-like water-conserving mechanism.^4^

To counteract osmotic diuresis and conserve water, the body increases medullary osmotic pressures by increasing urea production and accumulation. Under conditions of energy shortfall—eg, during persistent glucosuria—amino acids from skeletal muscle proteins are mobilized and degraded, providing the necessary nitrogen for urea generation and glucose formation via gluconeogenesis.^4^ In keeping with the aestivation hypothesis, SGLT2 inhibition increases glucagon concentrations and decreases insulin-to-glucagon ratios, uniquely prompting gluconeogenesis without glycogenolysis.^14^ Thus, muscle tissue is essentially sacrificed to support water conservation and glycemic control (Fig 1), reducing basal metabolic rate.^4^ Numerous studies have reported muscle loss with SGLT2 inhibition,^5^ but they had considerably shorter follow-up (median 0.5 years) than landmark trials (up to 2.4 years),^2^^,^^3^ questioning whether the full extent of muscle loss over prolonged periods of SGLT2 inhibition has been adequately captured. Clearly, the effects of SGLT2 inhibitors on muscle mass involve slow processes that affect long-term outcomes, which must be distinguished from their acute, muscle mass-independent effects.

### Understanding Acute Hemodynamic Responses to SGLT2 Inhibition

Initiating SGLT2 inhibitor therapy characteristically causes an acute, dose-dependent GFR dip of ∼5 mL/min/1.73 m^2^ on average (Fig 1),^11^^,^^13^^,^^15^ which is reversible on treatment cessation.^11^ This dip, which increases endogenous filtrations markers, stems from enhanced tubuloglomerular feedback because of inhibition of proximal tubular sodium reabsorption, leading to increased sodium delivery to macula densa cells located at the junction of the thick ascending loop of Henle and the distal tubule. The energy-intensive reabsorption of sodium by these cells increases ATP conversion to adenosine. Adenosine then binds to adenosine type 1 receptors on neighboring afferent arteriolar smooth muscle cells, causing afferent vasoconstriction that lowers glomerular hydrostatic pressure, thereby reducing GFR.^11^ Another important acute effect, also resulting from lowered glomerular hydrostatic pressure, is reduced albuminuria.^16^ As a robust risk marker for kidney disease progression and cardiovascular events,^17^ reducing albuminuria with SGLT2 inhibitors is expected to confer renoprotection. However, despite extensive evidence linking albuminuria to accelerated GFR decline and adverse clinical outcomes,^17^ it remains uncertain whether therapy-related albuminuria reductions directly mediate slowed GFR decline or rather reflect altered glomerular hemodynamics that independently confer renoprotection. The interpretation of therapy-related albuminuria reductions therefore warrants closer consideration.

### Interpreting Therapy-Related Reductions in Albuminuria

Insights from pivotal studies on renin-angiotensin-aldosterone system blockade provide important context. The fact that renoprotection in key trials with angiotensin-converting enzyme (ACE) inhibitors and angiotensin II type 1 receptor blockers (ARBs) was not solely dependent on systemic blood pressure reduction, but also related to changes in albuminuria, likely reflects that systemic arterial blood pressure and glomerular hydrostatic pressure can change independently.^18^ In this context, reduced albuminuria may reflect a favorable combination of decreased glomerular hydrostatic pressure and improved peritubular flow, making it an informative marker of hemodynamic change rather than a direct causal mediator of slowed GFR decline.

A comprehensive interpretation of albuminuria must also consider its relationship to oxygen delivery within the kidney parenchyma. The kidney medulla operates at physiologically low partial pressure of oxygen and is highly vulnerable to further reductions.^19^ This vulnerability is exacerbated in CKD, where kidney tissue oxygenation is markedly reduced.^20^ Such physiology likely explains why therapeutic strategies that increase peritubular flow (eg, ACE inhibitors and ARBs) show greater benefit in patients with proteinuria than in those without.^21^ Consistent with the 2024 Kidney Disease: Improving Global Outcomes (KDIGO) framework, patients who have both reduced GFR and elevated albuminuria are at disproportionately higher risk of CKD progression and adverse outcomes, because albuminuria reflects not only glomerular barrier injury but also microvascular dysfunction and impaired oxygen delivery.^8^ In turn, microvascular dysfunction and diminished oxygen reserve render the kidney more susceptible to ischemic or hypertonic injury, for example, during vomiting, diarrhea, or heat stress.^22^ In this view, interventions that reduce albuminuria likely do so by improving peritubular perfusion and oxygen delivery, thereby conferring proportionally greater protection. Accordingly, linking kidney hemodynamics to oxygenation reinforces the interpretation of albuminuria as a proxy of underlying kidney physiology rather than a direct causal mediator.

This hemodynamic-oxygenation framework also clarifies why pharmacologically distinct interventions can produce similar changes in albuminuria yet divergent effects on long-term GFR. ACE inhibitors and ARBs, on one hand, and nonsteroidal anti-inflammatory drugs and calcineurin inhibitors, on the other, all reduce glomerular hydrostatic pressure and albuminuria. However, ACE inhibitors and ARBs slow GFR decline, whereas nonsteroidal anti-inflammatory drugs and calcineurin inhibitors can accelerate it, especially at higher dosages. This paradox is best explained by differences in peritubular blood flow and oxygen delivery. Preferential efferent vasodilation with ACE inhibitors and ARBs increases oxygen supply, whereas afferent vasoconstriction with nonsteroidal anti-inflammatory drugs and calcineurin inhibitors reduces it. Both mechanisms reduce albuminuria, but only the former confers clear renoprotection. Thus, within the specific context of therapeutic interventions that alter kidney microvascular hemodynamics, albuminuria is best appreciated in the context of shifts in oxygen balance in the kidney rather than directly driving renoprotection.

Collectively, this hemodynamic-oxygenation framework challenges a causal role for albuminuria reduction in mediating renoprotection. For SGLT2 inhibitors, this uncertainty is substantiated by a recent mediation analysis of the Empagliflozin Cardiovascular Outcome Event Trial in Type 2 Diabetes Mellitus Patients–Removing Excess Glucose (EMPA-REG OUTCOME) trial, which identified changes in hematocrit and hemoglobin, rather than albuminuria, as the strongest mediators of the observed kidney benefits with empagliflozin.^23^ Interpreting the albuminuria reduction observed with SGLT2 inhibitors requires a careful consideration within the context of this framework. Acutely enhanced tubuloglomerular feedback following SGLT2 inhibition causes afferent vasoconstriction, which lowers glomerular hydrostatic pressure and peritubular flow. However, emerging evidence suggests that the precise vascular response to SGLT2 inhibition may differ by diabetes type, with typical afferent vasoconstriction predominating in type 1 diabetes, whereas efferent vasodilation appears more prominent in type 2 diabetes,^13^^,^^24^ likely reflecting greater preglomerular vascular stiffness and endothelial dysfunction in the latter condition.^24^ Nevertheless, these distinct hemodynamic adjustments likely converge on the common denominator of lowering glomerular hydrostatic pressure, albeit through different vascular routes. The mechanistic basis and clinical relevance of these divergent responses remain active areas of investigation, particularly in populations without comorbid diabetes, in whom the prevailing vascular response and glomerular dynamics may differ substantially.

Based on the accepted mechanism of afferent vasoconstriction, one would anticipate global tubular hypoxia during SGLT2 inhibition, particularly within the inner medulla, where the partial pressure of oxygen is physiologically lowest.^19^ However, experimental and imaging studies show a more nuanced picture in which cortical oxygenation increases because of reduced proximal tubular workload and energy demand, whereas medullary oxygenation decreases, owing to both reduced peritubular perfusion and increased tubular workload arising from the distally shifted sodium reabsorption.^20^^,^^25^ Given the absence of concomitantly increased mineralocorticoid activity,^26^ coupled with evidence of enhanced cortical oxygenation (in which the distal convoluted tubule resides), the distally shifted sodium is most plausibly reabsorbed in the thick ascending loop of Henle, located in the physiologically oxygen-deprived inner medulla.^19^

The resulting improvement in cortical oxygenation may confer local renoprotection, particularly in diabetes, in which proximal tubular glucose reabsorption operates at full capacity. However, the concomitantly intensified medullary hypoxia, evidenced by increased hemoglobin and hematocrit for as long as SGLT2 inhibition continues,^3^^,^^12^^,^^27^ warrants close monitoring, especially because this treatment strategy is increasingly applied to high-risk patients with advanced kidney impairment and reduced medullary reserve.^28^ Future mechanistic and clinical studies are eagerly awaited, because they may more definitively elucidate whether the observed reductions in albuminuria during SGLT2 inhibition primarily reflect favorable hemodynamic adaptation or concomitant shifts in regional oxygenation.

Beyond albuminuria, exploratory studies in patients with type 2 diabetes have reported consistent reductions in markers of tubular inflammation and injury during SGLT2 inhibition, suggesting potential nephron-level protective effects. Post-hoc analyses of the Canagliflozin Cardiovascular Assessment Study (CANVAS) demonstrated that canagliflozin reduced albuminuria as well as urinary excretion of monocyte chemoattractant protein 1 (MCP-1), tumor necrosis factor receptors 1 and 2, and kidney injury molecule 1 (KIM-1),^29^^,^^30^ with mediation analyses indicating that the reduction in urinary KIM-1 excretion was partly explained through sequential reductions in albuminuria and MCP-1.^29^ Similarly, a smaller short-term study reported that dapagliflozin reduced albuminuria as well as urinary excretion of interleukin-6 and KIM-1, with urinary MCP-1 excretion showing a nonsignificant numerical decline likely because of limited sample size.^31^

These observations integrate well with the hemodynamic-oxygenation framework discussed above and are consistent with broader mechanistic models of kidney protection by SGLT2 inhibitors that emphasize metabolic and oxygen-handling adaptations as central to renoprotection.^13^ Reductions in proximal tubular workload may improve cortical oxygenation by lowering energy demand,^20^^,^^25^ which, in turn, mitigates tubular inflammation and injury. Indeed, experimental and conceptual work has shown that MCP-1 expression increases sharply when peritubular flow and oxygen delivery decline,^22^ consistent with classical observations that hypoxia activates leukocyte adhesion, endothelial dysfunction, and chemokine signaling within the microcirculation of the kidney.^19^^,^^20^ Restoration of cortical oxygenation with SGLT2 inhibitors in type 2 diabetes may therefore explain the observed suppression of MCP-1 activity, reducing leukocyte recruitment and subsequent inflammatory injury, aligning molecular responses with improved oxygen balance.

However, such effects are probably most relevant in the setting of poorly controlled diabetes, in which proximal tubular glucose reabsorption operates at maximal capacity and cortical oxygen demand is highest. By contrast, comparable studies in nondiabetic CKD populations are lacking, leaving uncertain whether similar tubular marker changes occur when glucotoxicity and hyperfiltration are absent. Future mechanistic studies could therefore extend biomarker profiling to nondiabetic CKD to clarify whether these observed tubular benefits are disease-specific or represent a generalized adaptation to SGLT2 inhibition.

Altogether, these findings highlight the complexity of interpreting changes in endogenous filtration and risk markers in clinical studies. Because SGLT2 inhibitors also affect muscle mass, a proper understanding of the downstream effects on creatinine as established marker of GFR in trials is crucial. This understanding is not only a matter of scientific precision but also has implications for adherence to clinical research guidelines.

## Implications for Interpreting Creatinine-Based eGFR Endpoints

Regulatory bodies deem eGFRcr-based endpoints valid only if interventions do not alter muscle-derived creatinine generation.^9^ Given that SGLT2 inhibitors induce muscle loss and hence reduce creatinine generation,^4^^,^^5^ such interventions cause overestimation of eGFRcr (Fig 1). Therefore, SGLT2 inhibitor trials that used eGFRcr contravened the guideline stipulations and inadvertently inflated false-positive signals of renoprotection due to overestimated eGFRcr.^9^ This concern is underscored by findings of higher eGFRcr but unchanged muscle mass-insensitive cystatin C-based eGFR in patients with type 2 diabetes on SGLT2 inhibitors compared with matched controls.^32^ Although cystatin C—a housekeeping gene product—shows stable concentrations amid fluctuating muscle mass,^33^ its utility as a GFR marker in trials investigating the effects of SGLT2 inhibitors on kidney function equally merits caution because of SGLT2 inhibitor-related improved inflammatory status, reduced adiposity, and altered basal metabolism.^4^^,^^8^ Because cystatin C concentrations are positively related to inflammatory status, adiposity, and basal metabolic rate,^8^ SGLT2 inhibitors can reduce cystatin C concentrations by affecting these factors, irrespective of actual changes in GFR.

Consequences of overestimated GFR are serious and multifaceted. First, the observed renoprotection from SGLT2 inhibition is likely confounded by muscle loss—an argument that remains unchallenged because the long-term effects of SGLT2 inhibitors on GFR have never been confirmed with muscle mass-insensitive measures of GFR. Consequently, analyses with commonly used eGFRcr slopes as a surrogate for changing GFR should be re-evaluated.^9^

To illustrate the potential magnitude of this methodological bias, we conducted a quantitative estimation using representative data from the DAPA-CKD trial^2^ (Box 1^34^, ^35^, ^36^, ^37^, ^38^, ^39^). Assuming that eGFRcr unadjusted for body surface area approximates creatinine clearance, the observed between-group difference in annual change in eGFRcr in DAPA-CKD (estimated at −0.93 mL/min/1.73 m^2^) corresponds to a loss of approximately 0.45 kg of skeletal muscle per year if fully due to reduced creatinine generation. This equates to only ∼1%-2% of total-body muscle mass in an average adult,^39^ well within the range of muscle-mass losses reported in body-composition studies of SGLT2 inhibitors.^4^^,^^5^ Thus, even modest therapy-related reductions in muscle mass could plausibly account for much of the observed attenuation of eGFRcr decline over time, rather than reflecting genuine preservation of GFR.

Box 1 **Quantitative estimation of the magnitude of muscle mass loss required to meaningfully bias eGFRcr**

Using baseline statistics from the DAPA-CKD trial,^2^ specifically those for participants who received dapagliflozin, we estimated the extent of skeletal muscle mass loss required to fully explain the observed between-group difference in annual change in eGFRcr (−0.93 mL/min/1.73 m^2^) if the difference arose entirely from reduced creatinine generation rather than true GFR preservation.

A stepwise derivation is outlined as follows:

1. Relationship between creatinine and eGFRcr
  Creatinine clearance (*C*_*cr*_) is defined as:

$$\;C_{cr}=\frac{U_{cr}\times V}{P_{cr}}$$

where *U*_*cr*_ (in mg/dL) is the urinary creatinine concentration, *V* (mL/min) is the urine flow rate derived from the 24-hour urine volume, and *P*_*cr*_ (in mg/dL) is the plasma creatinine concentration. Rearranging gives the total creatinine excretion rate (CER, in mg/d):

$$\;\text{CER}=U_{cr}\times V=C_{cr}\times P_{cr}$$

We assume that eGFRcr unadjusted for body surface area (BSA), denoted ${\text{eGFR}}_{cr}^{*}$, is approximately equal to *C*_*cr*_:

$$\;C_{cr}≅{\text{eGFR}}_{cr}^{*}⟹\text{CER}≅{\text{eGFR}}_{cr}^{*}\times P_{cr}$$

2. Undoing the adjustment for BSA

The unadjusted ${\text{eGFR}}_{cr}^{*}$ is obtained by multiplying by the mean BSA:

$$\;{\text{eGFR}}_{cr}^{*}={\text{eGFR}}_{cr}\times \frac{\text{BSA}}{1.73}$$

The mean BSA at baseline is estimated using the Du Bois formula^34^:

$$\;BSA=0.007184\times H^{0.725}\times W^{0.425}$$

where *H* (cm) is height and *W* (kg) is weight. Using the mean weight of 81.5 kg and body mass index of 29.4 kg/m^2^ from DAPA-CKD, the mean height is:

$$\;H=100\sqrt{\frac{W}{\text{BMI}}}=100\sqrt{\frac{81.5}{29.4}}$$

Substituting gives:

$$\;\text{BSA}=0.007184\times {\left(100\sqrt{\frac{81.5}{29.4}}\right)}^{0.725}\times 81.5^{0.425}\approx 1.90\;m^{2}$$

Thus,

$$\;{\text{eGFR}}_{cr}^{*}={\text{eGFR}}_{cr}\times \frac{1.90}{1.73}=1.10\times {\text{eGFR}}_{cr}$$

3. Midpoint approach for average eGFRcr in DAPA-CKD

Because GFR declines gradually, we estimated the mean eGFRcr at the midpoint of the follow-up period (15 of 30 months) that was used to estimate the annual change in eGFRcr in DAPA-CKD using the estimated baseline eGFRcr (43.2 mL/min/1.73 m^2^) as well as annual eGFRcr slope (−2.86 mL/min/1.73 m^2^) in the dapagliflozin arm:

$$\;\bar{{\text{eGFR}}_{cr}}=43.2+\frac{15}{12}\times \left(-2.86\right)=39.6\;\text{mL}/\min/1.73\;m^{2}$$

This provides a representative average eGFRcr for downstream calculations, assuming linear change over time.

4. Cohort-weighted plasma creatinine concentration

The DAPA-CKD trial used the 2009 Chronic Kidney Disease Epidemiology Collaboration (CKD-EPI) equation^35^ to derive eGFRcr (published before the release of the race-free 2021 CKD-EPI equation^36^). Recognizing the problematic nature of race adjustment in clinical algorithms,^37^ we also derived $P_{cr}$ using the 2021 race-free CKD-EPI equation.

For both GFR-estimating equations, plasma creatinine was back-calculated separately for men and women as well as Black and non-Black subgroups, weighted by cohort proportions (mean age at 15 months of follow-up: 63.1 years; 32.9% women; 4.8% Black):

$$\;\bar{P_{cr}}=\sum_{g}w_{g}P_{cr,g}$$

This gave $\bar{P_{cr}}$ values of 1.82 mg/dL (2009 CKD-EPI equation) and 1.75 mg/dL (2021 CKD-EPI equation).

5. Annual change in CER
Daily CER (mg/d) is derived as:

$$\;\text{CER}={\text{eGFR}}_{cr}^{*}\times \bar{P_{cr}}\times 14.4$$

where the multiplier 14.4 converts clearance from mL/min to mg/d (1440 min/d divided by 100 mg/dL).

Substituting values using the CKD-EPI equations from 2009 (first value) and 2021 (second value):

$$\;\begin{array}{l} -0.93\times 1.10\times 1.85\times 14.4=26.8\;\text{mg}/d/y\\ -0.93\times 1.10\times 1.75\times 14.4=25.8\;\text{mg}/d/y \end{array}$$

6. Conversion to skeletal muscle mass

The relationship between daily CER (in g/d) and skeletal muscle mass (SM, in kg) is:

SM=k×CER

where *k* is the ‘creatinine equivalence’ (17.1 kg SM per g CER), here set at the midpoint of values of *k* observed for ad libitum diets.^38^

Differentiating and converting to grams per day gives:

$$\;{\Delta \text{SM}}_{year}=k\times \frac{{\Delta \text{CER}}_{year}}{1000}$$

Hence, using the CKD-EPI equations from 2009 (first value) and 2021 (second value):

$$\begin{array}{l} \;17.1\times \frac{26.8}{1000}=0.46\;\text{kg}\\ \;17.1\times \frac{25.8}{1000}=0.44\;\text{kg} \end{array}$$

**Interpretation**

A muscle mass loss of approximately 0.45 kg/y would be sufficient to explain the full 0.93 mL/min/1.73 m^2^ difference in annual change in eGFRcr between dapagliflozin and placebo in DAPA-CKD^2^ if the effect was entirely due to reduced creatinine generation. This corresponds to approximately 1%-2% of total muscle mass in an average adult,^39^ a biologically plausible change. These findings underscore that small, physiologically realistic shifts in muscle mass can meaningfully bias eGFRcr in clinical trials.

Ongoing muscle loss would therefore cause eGFRcr to present an overoptimistic view of true GFR. This could explain why eGFRcr slopes in the treatment compared with placebo arms continue to diverge throughout the trials (Fig 1), even as weight stabilizes because of reduced basal metabolic rate.^4^ Reduced basal metabolic rate results in a new steady state that stabilizes muscle mass at a new, lower baseline. Although therapy-related muscle loss can be substantial, it does not continue indefinitely but plateaus as the body adapts to therapy.

Second, effects of SGLT2 inhibition on “hard” endpoints such as dialysis initiation or transplantation might be susceptible to bias. Although nephrologists often emphasize initiating dialysis or recommending transplantation based on uremic complaints or fluid overload, in practice, declining eGFRcr plays a decisive role. This is reflected in historical trends toward earlier dialysis initiation and repeated cautions against relying on a predefined eGFR threshold without clinical context.^40^^,^^41^ Accordingly, although landmark trials of SGLT2 inhibitors have reported significant reductions in dialysis or transplantation, these findings should be interpreted with awareness that therapy-related changes in creatinine generation could influence both surrogate measures and the timing of clinical decisions. These interpretative considerations are particularly relevant when examining the reported event rates in landmark trials.

In the Dapagliflozin and Prevention of Adverse Outcomes in Chronic Kidney Disease (DAPA-CKD) trial,^2^ dialysis initiation or kidney transplantation occurred in 69 participants who received dapagliflozin versus 100 participants who received a matching placebo, corresponding to a hazard ratio of 0.66 (95% confidence interval, 0.49-0.90). Similarly, in The Study of Heart and Kidney Protection With Empagliflozin (EMPA-KIDNEY),^3^ these events occurred in 108 participants who received empagliflozin versus 158 participants who received a matching placebo (hazard ratio, 0.67; 95% confidence interval, 0.52-0.85). Importantly, SGLT2 inhibitors do not appear to increase the incidence of urgent dialysis initiation because of hyperkalemia, fluid overload, or overt uremic complications, which would be expected if the observed treatment benefit merely reflected deferred kidney replacement therapy. On the contrary, safety analyses from the Canagliflozin and Renal Events in Diabetes with Established Nephropathy Clinical Evaluation (CREDENCE),^42^ DAPA-CKD,^2^ and EMPA-KIDNEY^3^ trials did not indicate increased incidence of such adverse events in the active treatment arms, a finding consistent with the kaliuretic and hemodynamic effects of this drug class.^43^^,^^44^ These observations suggest that although part of the reduction in kidney replacement therapy may reflect genuine clinical benefit, particularly in the absence of increased emergent indications, some of the observed effect may still arise from delayed elective initiation in the context of lower creatinine generation and subsequent overestimation of eGFRcr. The contention, therefore, is not that such favorable effects were absent altogether, but that their interpretation may be complicated by the effects of SGLT2 inhibitors on creatinine generation. Specifically, it concerns long-term eGFRcr trajectories, not transient increases in creatinine concentrations or beneficial changes such as the reduced incidence of acute kidney injury observed in landmark SGLT2 inhibitor trials^2^^,^^3^^,^^42^ and in a recent individual participant data meta-analysis,^45^ because the latter primarily reflect hemodynamic and tubular adaptations rather than altered creatinine generation.

Moreover, the timing of dialysis or transplantation has direct implications for mortality, because these invasive procedures cause a period of excess mortality risk before long-term benefits accrue.^46^^,^^47^ If interventions bias eGFRcr readings upward and thereby delay such procedures, it could misleadingly manifest as survival benefits for these endpoints in trials. At the same time, although the incidence of urgent dialysis initiations has not been reported separately in landmark trials, it is noteworthy that no excess deaths due to kidney failure have been observed with SGLT2 inhibitors.^2^^,^^3^^,^^42^ This likely reflects genuine benefit from their kaliuretic and hemodynamic effects, reducing the risk of complications that can rapidly culminate in death if unaddressed.

## Recommendations for Future Research

Our concerns warrant a re-evaluation of the effects of SGLT2 inhibition on GFR. We emphasize that our critique does not dispute the benefits of SGLT2 inhibitors. Rather, we aim to highlight that their observed renoprotective effects require validation with muscle mass-independent measures. Definitive answers can only be provided by directly measuring GFR using exogenous filtration markers unaffected by treatment-related changes in muscle mass, inflammatory status, adiposity, and basal metabolic rate. Such procedures have been successfully employed in landmark trials such as the The African American Study of Kidney Disease and Hypertension (AASK),^21^ Modification of Diet in Renal Disease (MDRD),^48^ and Ramipril Efficacy In Nephropathy (REIN) studies.^49^ Alternatively, recognizing the infeasibility of such procedures, analyses with cystatin C-based eGFR, which is less influenced by muscle mass but potentially affected by other factors, could offer valuable insights.

In addition, there is a clear need for quantitative modeling studies that estimate the extent of muscle mass loss with therapies likely to influence it and, crucially, whether such losses meaningfully bias eGFRcr. Such work could be informed by:

• Longitudinal trial cohorts treated with SGLT2 inhibitors or other interventions that affect body composition;
• Stratification by baseline characteristics such as age, sex, and comorbid conditions (eg, diabetes and CKD);
• Use of reliable body composition measures (eg, height-indexed appendicular lean mass from dual-energy x-ray absorptiometry^6^^,^^50^ as surrogates of muscle mass) as well as surrogates of metabolically active muscle tissue (eg, height-indexed 24-hour creatinine excretion rate^10^ or creatine dilution test^51^). Also assess muscle strength (eg, grip strength^51^);
• Parallel assessment of cystatin C-based eGFR to disentangle creatinine-related artifacts from true GFR changes.

By combining GFR assessments, separately estimated using creatinine and cystatin C, with reliable muscle mass and strength measures, such studies could provide the quantitative context needed to judge whether observed kidney benefits are partly due to methodological bias or reflect genuine improvements in kidney physiology. We stress that this line of investigation is particularly pertinent in elderly patients in whom sarcopenia is both common and clinically consequential^6^; unlike younger individuals, older frail patients have limited to negligible capacity to regain lost muscle mass,^51^^,^^52^ which heightens the potential clinical impact of drug-induced changes in muscle metabolism.
